# Modulation of Radiative Heat Transfer at the Nanoscale via Topological Polaritons in Twisted van der Waals Crystals

**DOI:** 10.1002/nap2.70000

**Published:** 2026-01-27

**Authors:** Yang Hu, José Álvarez‐Cuervo, Enrique Terán‐García, Xiuquan Huang, Pablo Alonso‐González

**Affiliations:** ^1^ School of Power and Energy Northwestern Polytechnical University Xi'an Shaanxi China; ^2^ Department of Physics University of Oviedo Oviedo Spain; ^3^ Center of Research on Nanomaterials and Nanotechnology CINN (CSIC‐Universidad de Oviedo) El Entrego Spain

**Keywords:** α‐MoO_3_, near‐field radiative heat transfer, polaritons, topological transition, twisted stacks, van der Waals material

## Abstract

Twisted layers of α‐MoO_3_ support phonon polaritons whose propagation can be adjusted by the twist angle, a concept known as ‘twistoptics’. Although emergent in the field of nano‐optics, the application to heat transfer has lagged behind, particularly regarding near‐field radiative heat transfer (NFRHT), which is important for thermal management in nanodevices and remains insufficiently explored. Here, we report the role of twistoptics in NFRHT, demonstrating that the heat flux between two separated twisted α‐MoO_3_ bilayers can be monotonically increased by simply increasing the twist angle. Interestingly, this modulation is explained by the emergence of topological transitions from open (hyperbolic) to closed (elliptical) polaritonic dispersions. This phenomenon is further demonstrated by considering α‐MoO_3_ gapped trilayers, which show greater flexibility in regulating the NFRHT due to the emergence of a wider variety of topological transitions. Based on these findings, we propose an experimental scenario where the NFRHT between a nanoparticle and a closely spaced twisted α‐MoO_3_ bilayer can be modulated by a factor of 3 by simply adjusting the twist angle. This work provides theoretical guidance for the modulation of NFRHT using twistoptics, making an important step toward the development of twisted thermotics.

## Graphical abstract

Twisted α‐MoO_3_ layers support phonon polaritons whose propagation depends on the twist angle, enabling control of near field radiative heat transfer. Increasing the angle yields a monotonic rise in heat flux, driven by topological transitions from open hyperbolic to closed elliptical dispersions.

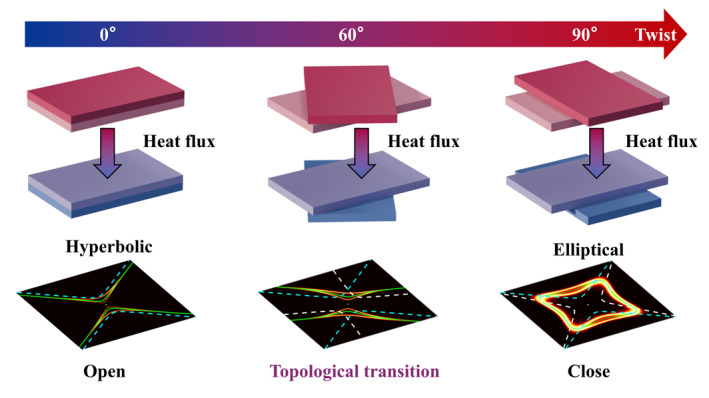

## 1 Introduction

Twisted stacks of van der Waals (vdW) crystals have emerged as an exceptional material platform for the discovery and controlled study of exotic optical phenomena [1, 2, 3, 4, 5, 6, 7, 8, 9]. A recent example is PhP canalisation in twisted bilayers and trilayers of the vdW semiconductor α‐MoO_3_ [10]. This phenomenon, characterised by the unidirectional propagation of the energy flux at the nanoscale, has its origin in the emergence of optical topological transitions, defined as the transition of polariton propagation from hyperbolic to circular (or elliptical), or vice versa [11, 12]. Beyond canalisation, recent studies show that twisted α‐MoO_3_ bilayers can also modulate hybrid polaritons, intrinsic chirality, Cherenkov radiation, and spontaneous emission, enriching the broader picture of twist‐engineered light–matter interactions [13, 14, 15, 16]. It is striking that the study of topological polaritons has predominantly focused on their propagative electromagnetic nature, while their role in radiative heat transfer, especially in the near field, remains largely unexplored. When the separation distance between two objects is comparable or smaller than the characteristic wavelength of thermal radiation, forward and backward evanescent waves can couple to each other, thus establishing pathways for photon tunnelling. This process gives rise to the near‐field radiative heat transfer (NFRHT) phenomenon, which can exhibit values significantly higher than those dictated by the blackbody limit stipulated by Planck's law of traditional radiation [17, 18, 19, 20, 21, 22, 23, 24, 25, 26, 27, 28, 29]. Importantly, this enhancement can be leveraged in various applications, such as thermophotovoltaic devices, thermal rectification, and thermal switches [30, 31, 32, 33, 34, 35, 36, 37, 38].

In particular, NFRHT between twisted structures separated by a vacuum gap has been addressed in the past [39, 40, 41, 42, 43, 44, 45, 46, 47, 48, 49], observing that the radiative heat flux shows a consistent decrease with increasing twist angle for monolayers (maximum when they are equally oriented) or a prominent enhancement near a given twist angle for bilayers. Such enhancement has been attributed to the excitation of surface plasmon polaritons undergoing a topological transition in their propagation. Despite these initial studies, key aspects for the optimisation of NFRHT using twisted layers remain to be elucidated, such as the precise role of the twist angle or the use of structures with more than two polaritonic thin layers.

In the present study, we introduce twisted van der Waals (vdW) multilayers composed of pairs of bilayers or pairs of trilayers separated by a vacuum gap as a material platform to efficiently modulate the heat transfer between them. Specifically, the results obtained show a marked monotonic increase of the NFRHT with increasing twist angle, reaching a maximum when the gapped bilayers, or gapped trilayers, are orthogonally aligned. By studying the resulting NFRHT as a function of layer thickness, we show that this finding originates from the occurrence of polaritonic topological transitions towards circular, or elliptical, dispersions (characterised by a closed isofrequency curve, IFC [1], in momentum space), which exhibit a higher density of optical states (DOS). Based on these results, we propose an experimental scheme in which the heat flux between a nanoparticle and a closely spaced twisted bilayer (50 nm) can be modulated up to a factor of 3 by simply varying the twist angle. These results pave the way for the development of nanodevices capable of efficient and reliable heat management, with potential applications in advanced thermal regulation systems.

**FIGURE 1:**
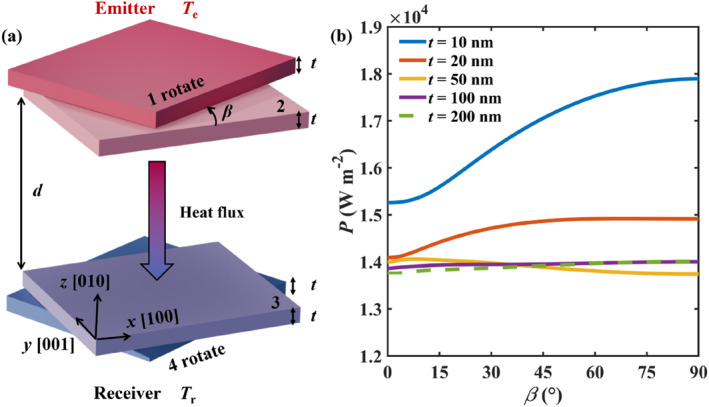
(a) Schematic representation of the NFRHT between a twisted *α*‐MoO_3_ bilayer (emitter) set at a temperature *T*_e_ = 320 K and a mirror‐symmetric structure (receiver) spaced a distance *d* = 50 nm apart and set at a temperature *T*_r_ = 300 K. The thickness of all *α*‐MoO_3_ layers is the same, as indicated by *t*. (b) NFRHT calculated as a function of twist angle *β* for different layer thicknesses *t*.

Figure 1a shows a schematic representation of the initially proposed NFRHT system based on gapped bilayers. The emitter and receiver, consisting of mirror symmetric twisted α‐MoO_3_ bilayers with thickness *t*, are considered in vacuum and separated a distance *d* = 50 nm. The bottom layers (labelled 1 and 4) are twisted simultaneously with an angle *β*. The radiative heat flux between the α‐MoO_3_ layers is calculated based on the fluctuation‐dissipation theory [17].

(1) $$P=\frac{1}{8\pi^{3}}\int_{0}^{\infty }\left[\Theta \left(\omega ,T_{e}\right)-\Theta \left(\omega ,T_{r}\right)\right]d\omega \int_{0}^{2\pi }\int_{0}^{\infty }\xi \left(\omega ,k,\phi \right)kdkd\phi ,$$

where $\Theta \left(\omega ,T\right)=\hbar \omega /\left[\exp\left(\hbar \omega /k_{B}T\right)-1\right]$ is the mean energy of a Planck oscillator at frequency *ω* and temperature *T*. ξ(ω,k,ϕ) is the energy transfer coefficient (ETC), which is given by the following equation:

(2) $$\xi \left(\omega ,k,\phi \right)=\left\{\begin{array}{l} \text{Tr}\left[\left(I-R_{12}^{*}R_{12}-T_{12}^{*}T_{12}\right)D\left(I-R_{34}^{*}R_{34}-T_{34}^{*}T_{34}\right)D^{*}\right],k<k_{0}\\ \text{Tr}\left[\left(R_{12}^{*}-R_{12}\right)D\left(R_{34}-R_{34}^{*}\right)D^{*}\right]e^{-2\left|k_{z}\right|d},k>k_{0} \end{array}\right.,$$

where $k=\sqrt{k_{x}^{2}+k_{y}^{2}}$ is the surface parallel wavevector, and *k*_0_ = *ω*/c is the wavevector in vacuum. $k_{z}=\sqrt{k_{0}^{2}-k^{2}}$ is the surface perpendicular wavevector, * signifies the complex conjugate, **R**_12/34_ and **T**_12/34_ represent the reflection/transmission coefficients of the emitter and receiver, and $D={\left(I-R_{12}R_{34}e^{2ik_{z}d}\right)}^{-1}$. Throughout this work, the coordinate axes are aligned with the main crystalline axes of the upper layer (labelled 2 and 3), that is, the *x* (*y*, *z*) axis is fixed to the [100] ([001], [010]) direction of the upper α‐MoO_3_ layer. Specifically, changes in twist angles are understood as a rotation of the lower layer with respect to the [100] direction of the upper α‐MoO_3_ layer.

Figure 1b shows the calculated radiative heat flux between the gapped *α*‐MoO_3_ bilayers as a function of the layer thickness *t* and the twist angle *β*. We observe that for the thicker layers (*t* = 100 nm and *t* = 200 nm), the radiative heat flux hardly varies with the twist angle. However, when the layer thickness is reduced to 20 nm, we observe a clear increase of NFRHT with *β* up to a value of 50°. This effect is most prominent when considering 10 nm thick layers, for which the NFRHT exhibits a clear maximum at *β* = 90° (corresponding to a 17% increase). These results convincingly demonstrate a twist angle‐dependent NFRHT for thin vdW layers, opening the door to efficient heat transfer control. Note that these results differ significantly from previous calculations on purely 2D material platforms, such as graphene gratings [38], which show the enhancement effect of the topological transitions of the surface state at the photonics magic angle.

**FIGURE 2:**
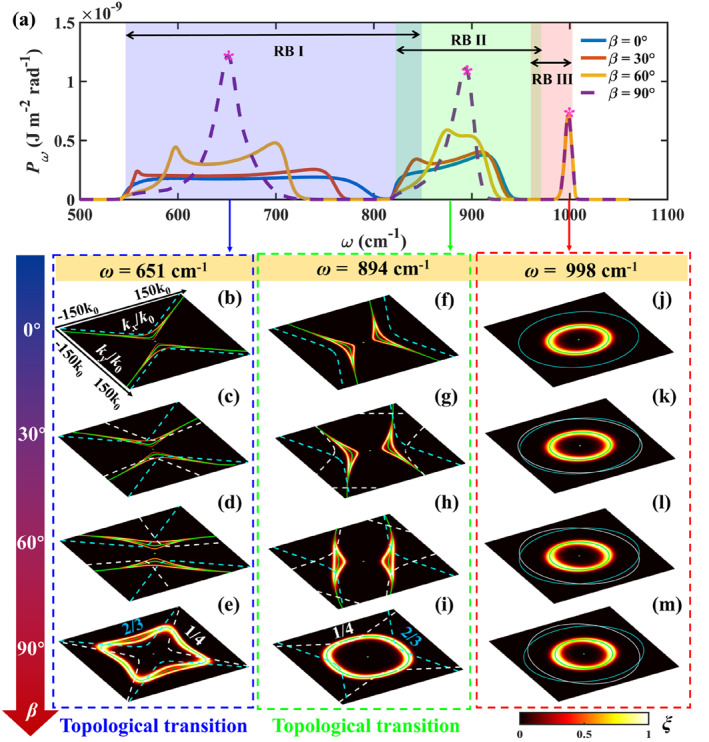
(a) NFRHT as a function of frequency for twist angles *β* = 0°, *β* = 30°, *β* = 60°, and *β* = 90° (blue, red, orange, and violet curves, respectively). The thicknesses *t* of both α‐MoO_3_ layers are 10 nm. (b–m) Energy transfer coefficient ξ(ω,k,ϕ) in *k*‐space for *β* = 0°, *β* = 30°, *β* = 60°, *β* = 90° (row 1–4, respectively) at frequencies *ω* = 651 cm^−1^ (b–e), *ω* = 894 cm^−1^ (f–i), and *ω* = 998 cm^−1^ (j–m). The green lines indicate the IFCs of the bilayer α‐MoO_3_. The cyan and white dashed lines indicate the IFCs of the top layer (i.e., without twist) and bottom layer (with twist) of the twisted bilayer α‐MoO_3_.

To better explore the physical origin of these results, particularly in the case of thin layers, we plot the NFRHT power density as a function of frequency, *P*_*ω*_. The resulting graphs for *t* = 10 nm (Figure 2a) show high values in three well‐defined spectral bands (labelled I, II, and III), which correspond exactly to those in which *α*‐MoO_3_ exhibits negative permittivity [50] (normally referred to as Reststrahlen bands, RB) and, therefore, supports surface PhPs. Interestingly, the spectral distribution of *P*_*ω*_ varies significantly with *β* within RBI and RBII. Specifically, it evolves from a relatively constant value for *β* = 0° to show a double peak function for *β* = 30° and 60° and, finally, a maximum for *β* = 90° (at frequencies of 651 and 894 cm^−1^ in RB I and RB II, respectively). Notably, the spectral concentration at the maxima for *β* = 90° is accompanied by an increase in the power density of up to a factor of 7.2. On the other hand, *P*_*ω*_ remains practically unchanged within RB III, showing in all cases a single peak at *ω* = 998 cm^−1^.

To interpret these results, we calculate the energy transfer coefficient ξ(ω,k,ϕ) (defined in Equation (2)) as a function of twist angle *β* and frequency (contour plots in Figure 2b–m). Note that this coefficient takes values in the range of 0–1 and accounts for p‐polarised waves (the contribution from *s*‐polarised waves is negligible). For the maximum of *P*_*ω*_ at *ω* = 651 cm^−1^, that is, within the RBI, we observe that ξ shows a hyperbolic contour that transitions to a square‐like curve as *β* increases, accompanied by a sharp increase in intensity. Interestingly, for *β* = 60°, the propagation of hyperbolic PhPs, indicated by their isofrequency contour IFC (green line in Figure 2d), becomes highly directional or canalised [1, 2, 3, 4] (flattened IFC). This polariton propagation enhances the radiative heat flux (colour plot) along a narrow angular region in *k*‐space. When *β* = 90°, the distribution of energy transfer coefficients in *k*‐space takes on a quasi‐square shape, as shown in Figure 2e. The value of energy transfer coefficients approaches 1, indicating highly efficient radiative heat flux transfer through hyperbolic PhPs between the pairs of bilayers. Furthermore, the energy transfer coefficients cover a broader range in *k*‐space compared to other twist angles, resulting in the highest radiative heat flux after integration. However, the total near‐field radiative heat flux is given by integrating the spectral heat flux over all frequencies and all parallel wavevectors. Thus, even though *ξ* at the resonance frequency changes significantly, that resonance band contributes only a portion of the full spectral integral. This same behaviour of *ξ* as a function of *β* is observed for the maximum of *P*_*ω*_ at *ω* = 894 cm^−1^, that is, within RB II, as shown in Figure 2f–i. As the twist angle increases, hyperbolic PhPs undergo a topological transition from an open (hyperbolic) to a closed (elliptical) IFC (green lines), resulting in a gradual enhancement of the heat flux. Although hyperbolic contours are often expected to outperform elliptical ones in NFRHT, the comparison is subtle and strongly parameter‐dependent. As shown by Liu and Zhang [51], losses and geometric cutoffs limit high‐*k* contributions, so hyperbolicity offers no universal benefit. Because ref. [51] compares different geometries, its cases are not directly comparable. In our study, only the twist angle varies, allowing a fair comparison between hyperbolic and elliptical regimes.

In sharp contrast, for the maximum of *P*_*ω*_ at *ω* = 998 cm^−1^, the energy transfer coefficient in *k*‐space does not undergo any topological transition as a function of the twist angle, resulting in a minimal variation of the total heat flux (Figure 2j–m).

Taken together, these results unambiguously demonstrate: (i) the appearance of a topological transition in gapped bilayers as a function of the twist angle and (ii) that this transition involves a modulation and eventual enhancement of ξ for a closed contour, which explains the sharp increase in *P*_*ω*_ (Figure 2a). Note that the same conclusions can be derived when considering bulky bottom media, as shown in Figures S2 and S3. It is important to note that the mechanism in plate–plate systems differs fundamentally from plate‐mediated nanoparticle interactions. For planar slabs, heat transfer is obtained by integrating the energy transmission coefficient over *k*‐space, whereas for nanoparticles above a plate, it is mainly governed by mode matching between the plate's polariton dispersion and the particles' positions [44, 49]. Consequently, a twist‐induced topological change affects NFRHT in a distinct manner.

Interestingly, Figure 2b–m reveals two separated branches of *ξ* at low momentum values. This observation seems to indicate the formation of two distinct polaritonic modes as a result of the electromagnetic coupling between the gapped bilayers (note that surface polaritons decay exponentially in the direction perpendicular to their propagation and this decay is proportional to 1/*k*). This is corroborated by observing that the IFCs for isolated twisted bilayers (green lines) lie between these branches, indicating that the origin of the latter is rooted in the presence of, and therefore coupling with, another twisted bilayer. The formation of symmetric–antisymmetric coupled modes has been reported previously for the case of surface polaritons in SiC [52]. However, to the best of our knowledge, there are no reports of such coupled modes for the case of hyperbolic volume polaritons, nor of their dependence on the twist angle.

**FIGURE 3:**
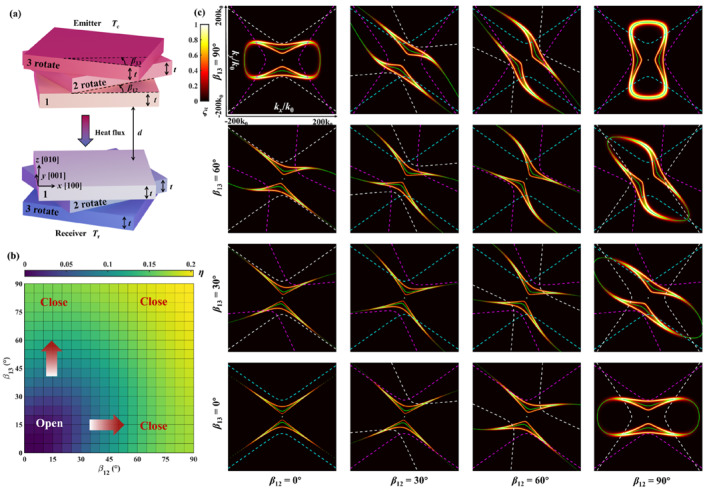
(a) Schematic representation of the NFRHT between a twisted α‐MoO_3_ trilayer (emitter) set at a temperature *T*_e_ = 320 K and a mirror‐symmetric structure (receiver) separated by a distance *d* = 50 nm and set at a temperature *T*_r_ = 300 K. The thickness of all α‐MoO_3_ layers is the same (*t* = 5 nm). (b) The normalised NFHT varies with twist angles *β*_12_ and *β*_13_. The thicknesses of both *α*‐MoO_3_ layers are 5 nm. (c) The energy transfer coefficients distribution in wavevector space. The frequency is *ω* = 633 cm^−1^. The twist angles are *β*_13_ = 0°, *β*_13_ = 30°, *β*_13_ = 60°, *β*_13_ = 90° corresponding to rows 1–4, and *β*_12_ = 0°, *β*_12_ = 30°, *β*_12_ = 60°, *β*_12_ = 90° corresponding to columns 1–4. The green lines are the IFCs of the *α*‐MoO_3_ trilayer. The cyan, white, and magenta dashed lines are the IFCs of the top layer (without twist), middle layer (with twist), and bottom layer (with twist) in the twisted trilayer.

To potentially expand the capabilities of twisted gapped stacks for controlling NFRHT, we further study α‐MoO_3_ gapped trilayers (Figure 3a), which has been shown to exhibit a large variety of polaritonic topological transitions [2]. As before, we consider layers of the same thickness *t*, whereas the twist angles, *β*_12_ and *β*_13_, are defined now between the [001] crystal direction of the top layer and the [001] crystal direction of the middle and bottom layers, respectively. By calculating the normalised radiative heat flux *η* [defined as (*P* − *P*_min_)/*P*_min_] between the trilayers (Figure 3b), we observe a clear dependence on both twist angles. Specifically, *η* is minimal for *β*_12_ = *β*_13_ = 0°, and reaches a maximum for *β*_12_ = *β*_13_ = 90°, with an overall enhancement of more than 20%. To explain this result, we plot in Figure 3c the energy transfer coefficient ξ in *k*‐space as a function of twist angles for the maximum of *P*_*ω*_ (see Figure S5) at *ω* = 633 cm^−1^. For the minimum of *η* at *β*_12_ = *β*_13_ = 0°, the contour of ξ is hyperbolic, corresponding to the excitation of hyperbolic PhPs as shown by the IFC contour (green lines). As the twist angle increases, ξ and the polaritonic IFC gradually transition toward a closed contour. Specifically, for the three cases corresponding to *β*_12_ = 0° and *β*_13_ = 90°, *β*_12_ = 90° and *β*_13_ = 0°, *β*_12_ = 90° and *β*_13_ = 90°, ξ exhibits closed elliptical contours, which are similar to the polaritonic IFCs previously observed for twisted trilayers [2]. More importantly, in these three cases ξ appears noticeably broader in *k*‐space and exhibits a maximum value (close to 1), which in turn leads to an enhanced radiative heat flux.

**FIGURE 4:**
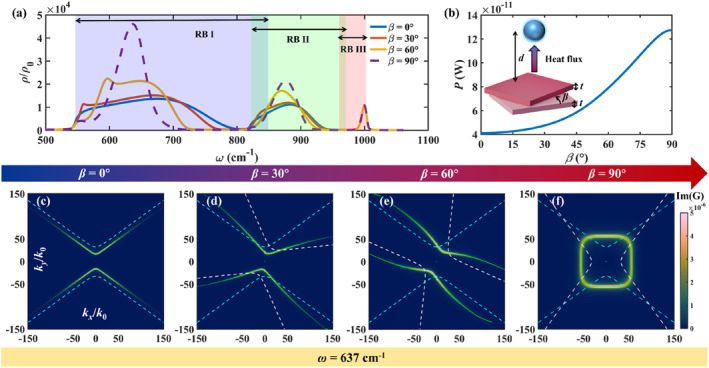
(a) LDOS of the α‐MoO_3_ bilayer as a function of frequency for different twist angles *β* = 0°, *β* = 30°, *β* = 60°, and *β* = 90° (blue, red, orange, and violet curves, respectively). (b) NFRHT between the α‐MoO_3_ bilayer and a NP as a function of twist angle. Inset: schematic representation of the system considered involving a twisted *α*‐MoO_3_ bilayer, set as emitter at a temperature *T*_e_ = 320 K, and a NP, set as receiver at a temperature *T*_r_ = 300 K, separated by a distance *d* = 50 nm. The thickness of all *α*‐MoO_3_ layers is the same (*t* = 5 nm). Imaginary part of the Green's function distribution in *k*‐space at *ω* = 637 cm^−1^ for *β* = 0° (c), *β* = 30° (d), *β* = 60° (e), *β* = 90° (f). The green lines indicate the IFCs of the *α*‐MoO_3_ bilayer. The cyan and white dashed lines indicate the IFCs of the top layer (without twist) and bottom layer (with twist) of the twisted bilayer.

To further highlight the potential of topological transitions in twisted stacks to modulate the NFRHT, we study the case of an α‐MoO_3_ bilayer in close proximity to a nanoparticle (NP). First, we calculate the spectral distribution of the local density of states (LDOS) of the α‐MoO_3_ bilayer for different twist angles (Figure 4a). We observe that it closely resembles that of the spectral radiative heat flux shown in Figure 2a, varying significantly with the twist angle and showing a strong peak in the RB I at *ω* = 637 cm^−1^. Next, we consider a NP with a strong LDOS response at this maximum, which is obtained by designing its dielectric function.

(3) $$\varepsilon \left(\omega \right)=1-\frac{\omega_{p}^{2}}{\omega^{2}+i\Gamma \omega }.$$

Specifically, we ensure that the NP resonance at *ε*(*ω*) = −2 coincides spectrally with the maximum of LDOS in the bilayer. Disregarding losses, this occurs for *ω*_*p*_ = 1103 cm^−1^ and Γ = 0.01*ω*_*p*_. Finally, we calculate the NFRHT between the bilayer and the NP for a separation distance of 50 nm (Figure 4b). We observe that the total radiative heat flux *P* increases monotonically with the twist angle, reaching a value up to 3 times higher for *β* = 90° compared to *β* = 0°. To investigate the mechanism of this large enhancement, we examine the imaginary part of the Green's function (proportional to the radiative heat flux) for the bilayer‐NP system in *k*‐space (at the resonance frequency). The Green's function can be obtained from the following equation [53]:

(4) $$G_{k}\left(\omega ,k_{x},k_{y}\right)=e^{ik_{z}z}\left[\left(r_{\text{ss}}e_{s+}+r_{\text{ps}}e_{p+}\right)e_{s-}^{T}+\left(r_{\text{sp}}e_{s+}+r_{\text{pp}}e_{p+}\right)e_{p-}^{T}\right],$$

where $e_{s\pm }={\left[\begin{array}{ccc} \frac{k_{y}}{k_{0}} & \frac{-k_{x}}{k_{0}} & 0 \end{array}\right]}^{T}$ and $e_{p\pm }=\frac{-1}{k_{0}}{\left[\frac{\pm k_{z}k_{x}}{k_{0}}\frac{\pm k_{z}k_{y}}{k_{0}}-\sqrt{k_{x}^{2}+k_{y}^{2}}\right]}^{T}$.

The resulting plots are shown in Figure 4c–f. When *β* = 0°, Im(G) shows a hyperbolic contour with upper and lower openings, closely aligning with the polaritonic IFC of the α‐MoO_3_ bilayer (dashed lines), indicating the direct relation between heat transfer and the excitation of hyperbolic PhPs. As the twist angle increases, Im(G) also increases. When *β* = 60°, the two hyperbolic branches of the polaritonic IFC are almost parallel at large wavevectors, resembling a canalisation phenomenon [46]. Finally, when *β* = 90°, Im(G) shows a closed contour resembling a circle, and the maximum magnitude. These results corroborate the key role of topological transitions for achieving modulation and eventual enhancement of NFRHT in twistoptics.

In conclusion, our results demonstrate the potential of twisted van der Waals layers to modulate and enhance the radiative heat flux at the nanoscale. These capabilities are explained by the emergence of polaritonic topological transitions (from hyperbolic to closed IFCs) as a function of the twist angle, as demonstrated for gapped bilayers and trilayers of the anisotropic vdW materials α‐MoO_3_. Based on these findings, we propose an experimental setup in which the heat flux between a nanoparticle and nearby twisted α‐MoO_3_ bilayer can be modulated up to a factor of 3. Our findings on topological transitions in NFRHT are broadly relevant to materials supporting in‐plane hyperbolic polaritons. The effect depends on anisotropy, losses, and geometry, and should occur in the infrared, where thermal radiation is most significant. Overall, this work introduces twistoptics as an efficient route for heat management applications.

## Author Contributions

**Yang Hu:** methodology, data curation, investigation, validation, visualization, writing – original draft, funding acquisition. **José Álvarez‐Cuervo:** investigation, writing – review & editing. **Enrique Terán‐García:** visualization, writing – review & editing. **Xiuquan Huang:** supervision, visualization, writing – review & editing. **Pablo Alonso‐González:** investigation, visualization, funding acquisition, supervision, writing – review & editing.

## Conflicts of Interest

The authors declare no conflicts of interest.

## Supporting information


Supporting Information S1

## Data Availability

Data will be made available at a reasonable request.
