# Supplementary material for: Modulation of Radiative Heat Transfer at the Nanoscale via Topological Polaritons in Twisted van der Waals Crystals
Source: Nanophotonics. 2026 Jan 27;15(2):e70000. doi: 10.1002/nap2.70000 (PMC12964987; doi:10.1002/nap2.70000)
Supplement: Supplementary file 1 — Supporting Information S1 [file NAP2-15-e70000-s001.docx]

**Supporting Information for:**

**Modulation of radiative heat transfer at the nanoscale via topological polaritons in twisted van der Waals crystals**

Yang Hu^1,2*^, José Álvarez-Cuervo^2,3^, Enrique Terán-García^2,3^, Xiuquan Huang^1,*^, and Pablo Alonso-González^2,3,*^

^1^School of Power and Energy, Northwestern Polytechnical University, Xi’an 710072, Shaanxi, P. R. China

^2^Department of Physics, University of Oviedo, Oviedo 33006, Spain

^3^Center of Research on Nanomaterials and Nanotechnology, CINN (CSIC-Universidad de Oviedo), El Entrego 33940, Spain

*^*^Corresponding author: Yang Hu (hu_yang@mail.nwpu.edu.cn), Xiuquan Huang (xiuquan_huang@nwpu.edu.cn) and Pablo Alonso-González (*[*pabloalonso@uniovi.es*](mailto:pabloalonso@uniovi.es)*)*

**Section 1.** **Effect on the radiative heat transfer of the layer thickness and separation distance of gapped α-MoO_3_ bilayers.**

The spectral radiative heat flux *P_ω_* for a layer thickness *t*=200 nm is shown in **Fig. S1(a)**. We observe that *P_ω_* remains nearly constant at different twist angles, with a sharp peak at *ω*=968 cm^-1^, which is attributed to the excitation of Dyakonov surface waves. The frequency-dependent oscillations arise from the excitation of higher-order hyperbolic modes. **Figs. S1(b-m)** shows the energy transfer coefficient *ξ* in *k*-space, showing its variation with the twist angle and frequency. The asymptotes of the hyperbolic isofrequency contours (IFCs) of phonon polaritons in the bilayers are also shown as blue dashed lines. For *ω*=637 cm^-1^, *ξ* shows a topological transition, but for a very limited range of wavevectors (<20 *k*/*k*_0_), which results in a negligible effect on NFRHT, as can be seen in **Figs. S1(f-i)**. As the separation between the emitter and receiver increases, modes at small wavevectors become dominant. Consequently, when the thickness of each layer is 200 nm, the topological transition still has a pronounced impact on the radiative heat transfer. For *ω*=968 cm^-1^, the IFC remains closed while varying the twist angle, i.e., without any polaritonic topological transition occurring, which results in minimal changes in the NFRHT.


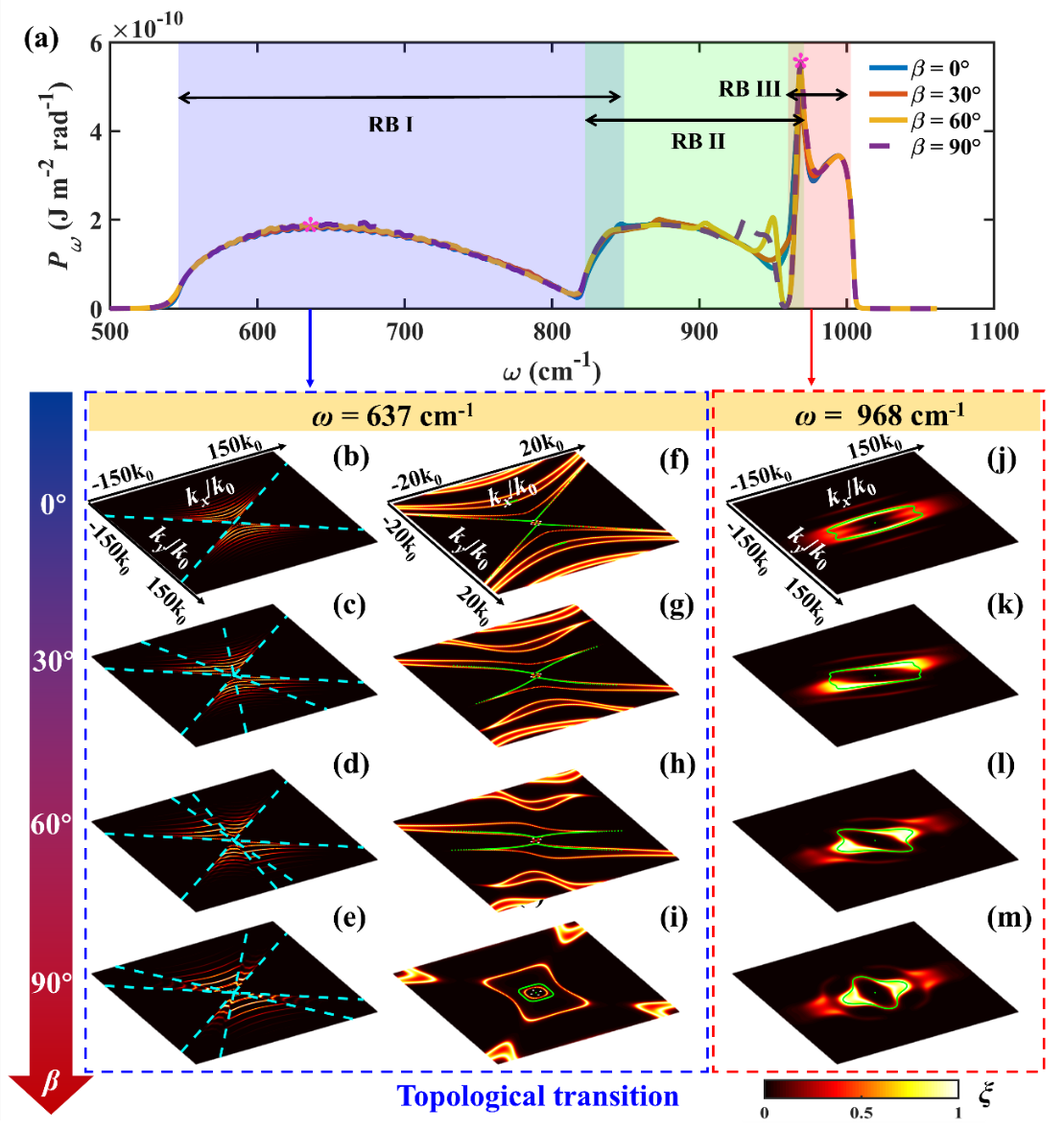


**Fig. S1. (a)** NFRHT as a function of frequency for different twist angles *β* = 0°, *β* = 30°, *β* = 60°, *β* = 90° (blue, red, orange, and violet curves, respectively). The thicknesses of both α-MoO_3_ layers are 200 nm. The in *k*-space for *β* = 0°, *β* = 30°, *β* = 60°, *β* = 90° (row 1 to 4, respectively) at frequencies *ω* = 637 cm^-1^ **(b-i)**, and *ω* = 968 cm^-1^ **(j-m)**. The blue lines indicate the asymptotes of hyperbolic phonon polaritons. The green lines indicate the IFCs of the bilayer α-MoO_3_.

**Section 2.** **NFRHT between gapped α-MoO_3_ bilayers for bulky bottom layers.**

In this section, we examined the variation of the NFRHT with the twist angle when the outer layers in the twisted stacks (1 and 4) are very thick (bulky) and the inner ones (2 and 3) are thin and vary in thicknesses *t*. For *t*=10 nm, we observe that the NFRHT increases by 17% as the twist angle increases from 0° to 90° (**Fig. S2(b)**). The trend observed resembles that in **Fig. 1(b)**, suggesting that the NFRHT primarily depends on the thickness of the outer layers. For and intermediate thickness *t*=50 nm, the NFRHT firstly increases and then decreases with the twist angle, showing a maximum at around *β*=20º. Finally, for *t*=200 nm, the radiative heat flux hardly changes with the twist angle.


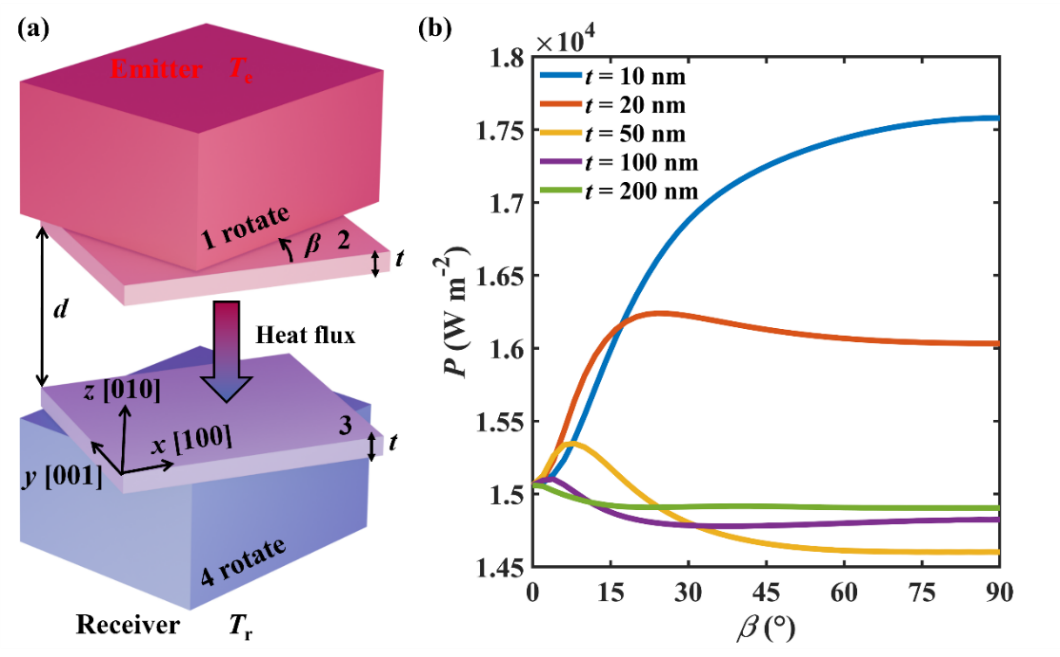


**Fig. S2(a)** Schematic representation of NFRHT between bilayer α-MoO_3_. The thickness of the top layer is the same, as indicated by *t*. The bottom parts (1 and 4) are bulk. The remaining parameters are set the same as in Fig. 1(a). **(b)** Calculated NFHT as a function of twist angle for different layer thicknesses *t*.

To explore the mechanism behind such variation in the radiative heat flux, we investigated its spectral dependence for different thicknesses (**Fig. S3(a)**). For *t* = 10 nm, *P_ω_* reaches a maximum at *ω*=647 cm^-1^, which is attributed to the enhancement of the radiative heat flux due to the presence of a topological transition—similar to the previous analysis. The variation of *P_ω_* as a function of twist angle decreases as the thickness of the inner layers increases, particularly within RBⅠ. Additionally, the contribution of surface modes (around *ω* = 968 cm^-1^) to *P_ω_* increases gradually with the thickness of the inner layers.

The underlying physics of the radiative heat flux enhancement at *ω*=647 cm^-1^ is illustrated by the variation of *ξ* in **Figs. S3(b-e)**. When *β* = 0°, hyperbolic phonon polaritons can be excited both in the upper and lower regions in *k*-space, as shown by the polaritonic IFCs (dashed lines). However, as the top layer rotates to *β* = 30°, the hyperbolic IFC in the bilayer flattens, giving rise to canalized propagating of polaritons along one direction, which is reflected in a similar shape for *ξ*. For *β* = 60° *ξ* shows a pinwheel pattern and for *β* = 90° an “8-like” shape. Interestingly, in this latter case both the intensity and the range in *k*-space of *ξ* are significantly enhanced compared to the case for *β* =0°, leading to a larger radiative heat flux.


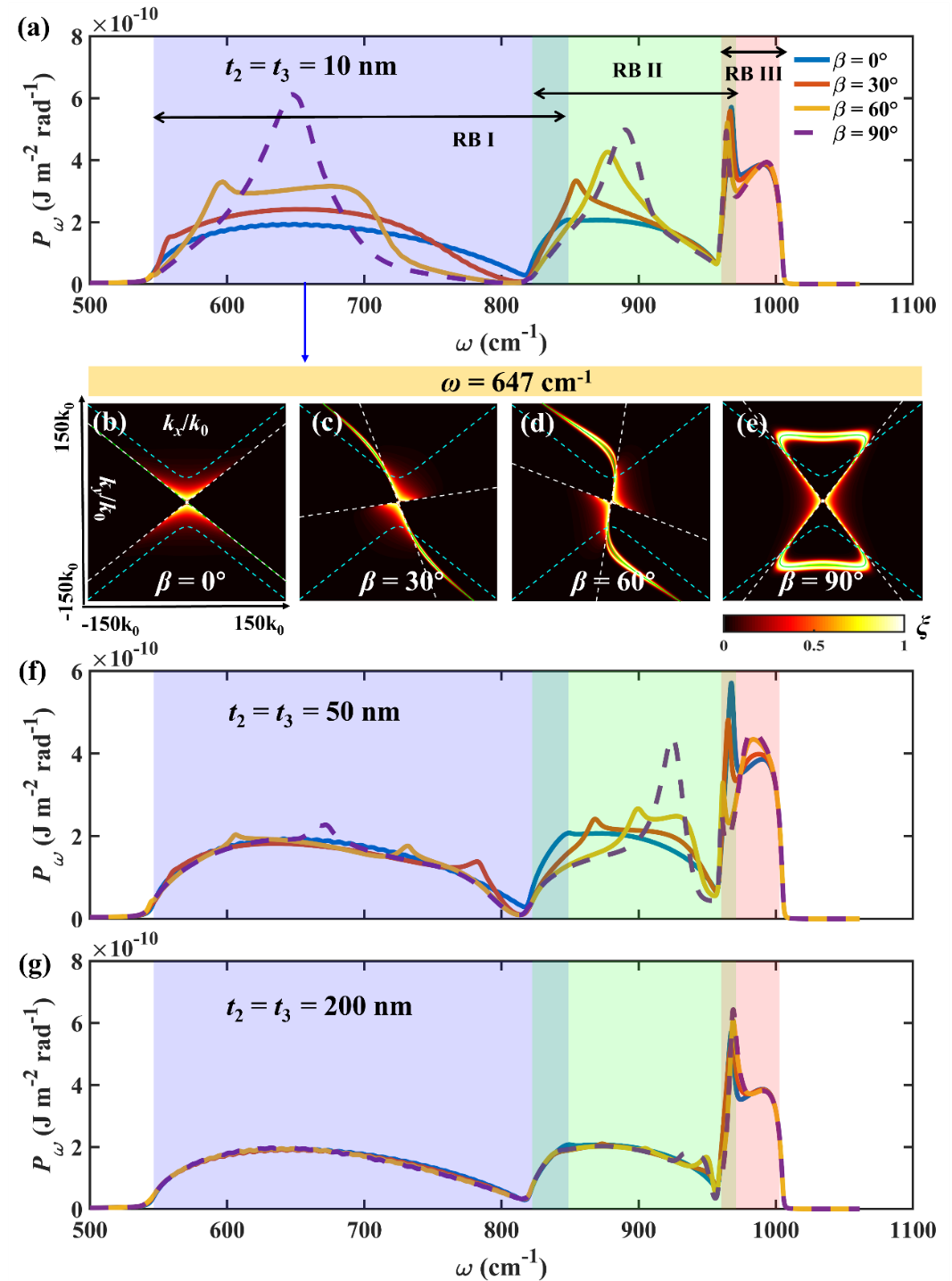


**Fig. S3.** NFRHT as a function of frequency for different twist angles *β* = 0°, β = 30°, *β* = 60°, and *β* = 90° (blue, red, orange, and violet curves, respectively). The thicknesses of the inner layers are 10 nm **(a)**, 50 nm **(f)**, and 200 nm **(g)**. The outer layers are considered as bulk. **(b-e)** Energy transfer coefficient in *k*-space for *t*=10 nm and *β* = 0°, 30°, 60°, and 90°, respectively. The frequency is fixed to the maximum at *ω* = 647 cm^-1^. The green lines indicate the IFCs of the α-MoO_3_ bilayer. The cyan and white dashed lines indicate the IFCs of the inner layer (with no rotation) and outer layer (with rotation), respectively.

**Section 3.** **Effect of the separation distance in the NFRHT between** **gapped α-MoO_3_ bilayers.**

The effect of the separation distance *d* in the NFRHT is shown in **Fig. S4**. We observe a rapid decay of NFRHT with increasing *d*, which is attributed to the fact that Eq. (2) has an exponential term when calculating the contribution of evanescent waves. Furthermore, for *t*=10 nm we observe an enhancement of the NFRHT regardless of *d*. This is in sharp contrast to the cases for thicker layers, *t*=50 nm and *t*=100 nm, for which there is a minimal variation of the NFRHT as a function of twist angle.


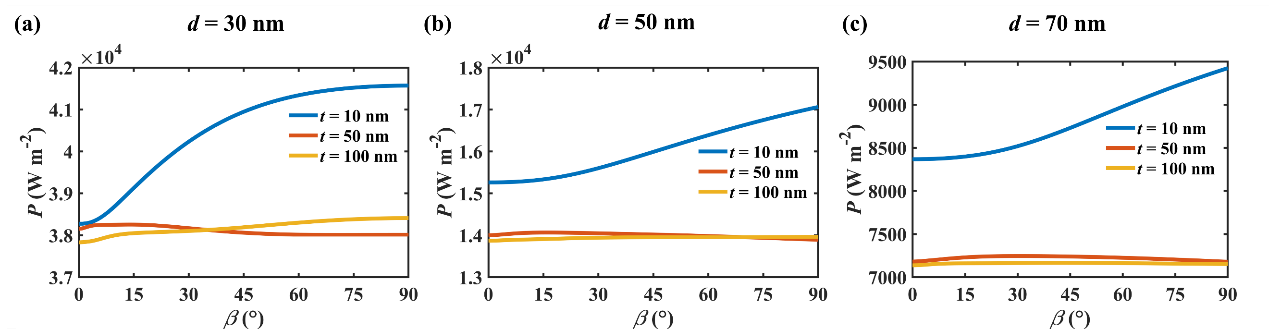


**Fig. S4.** **(a-c)** NFRHT as a function of separation distance *d* between gapped α-MoO_3_ bilayers for *d*=30 nm, *d*=50 nm, and *d*=70 nm, respectively.

**Section 4.** **Near-field radiative heat flux as a function of frequency for gapped α-MoO_3_ trilayers.**

The near-field radiative heat flux as a function of frequency, *P_ω_*, for gapped α-MoO_3_ trilayers varies with the twist angle between the layers *β*_12_ and *β*_12_ as shown in **Fig. S5**. Significant variations with the twist angle are observed in both RBⅠ and RBⅡ spectral regions. In the main manuscript, we analyze the energy transmission coefficient at the maximum of *P_ω_* in the RBⅠ (*ω* = 633 cm⁻¹).


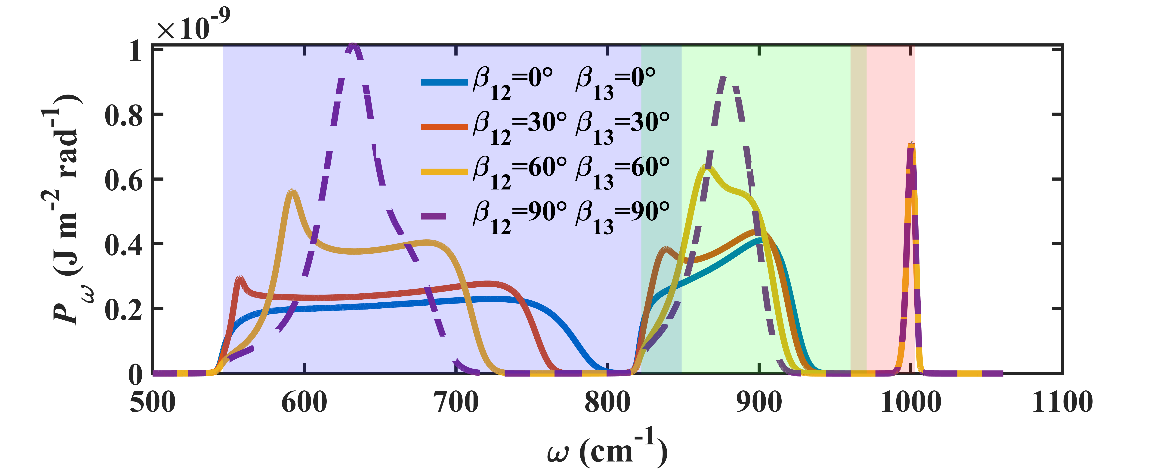


**Fig. S5.** NFRHT between gapped α-MoO_3_ trilayers as a function of frequency for twist angles *β*_12_ = 0°, *β*_13_ = 0°, *β*_12_ = 30°, *β*_13_ = 30°, *β*_12_ = 60°, *β*_13_ = 60°, and *β*_12_ = 90°, *β*_13_ = 90° (blue, red, orange, and violet curves, respectively). The thicknesses *t* of both α-MoO_3_ layers are 5 nm.

**Section 5. Near-field radiative heat flux as a function of frequency for different twist angles when the thickness of each layer is 50 nm.**

For a layer thickness of 50 nm (Fig. S6(a)), the spectral distribution of NFRHT shows a weaker dependence on twist angle compared to thinner layers, indicating a reduced effect of the topological transition. This can be understood from *ξ* distribution (Fig. S6 (b–e)), where the transition occurs only within a limited wavevector range (*k* < 100*k*_0_). In RB Ⅱ, the double-peak structure gradually evolves into a single peak as the twist angle increases from 0° to 90°, leading to a competition between their intensities. For the 50 nm case, the two peaks at 0° (836 cm⁻¹ and 952 cm⁻¹) are notably suppressed, resulting in lower integrated heat transfer.


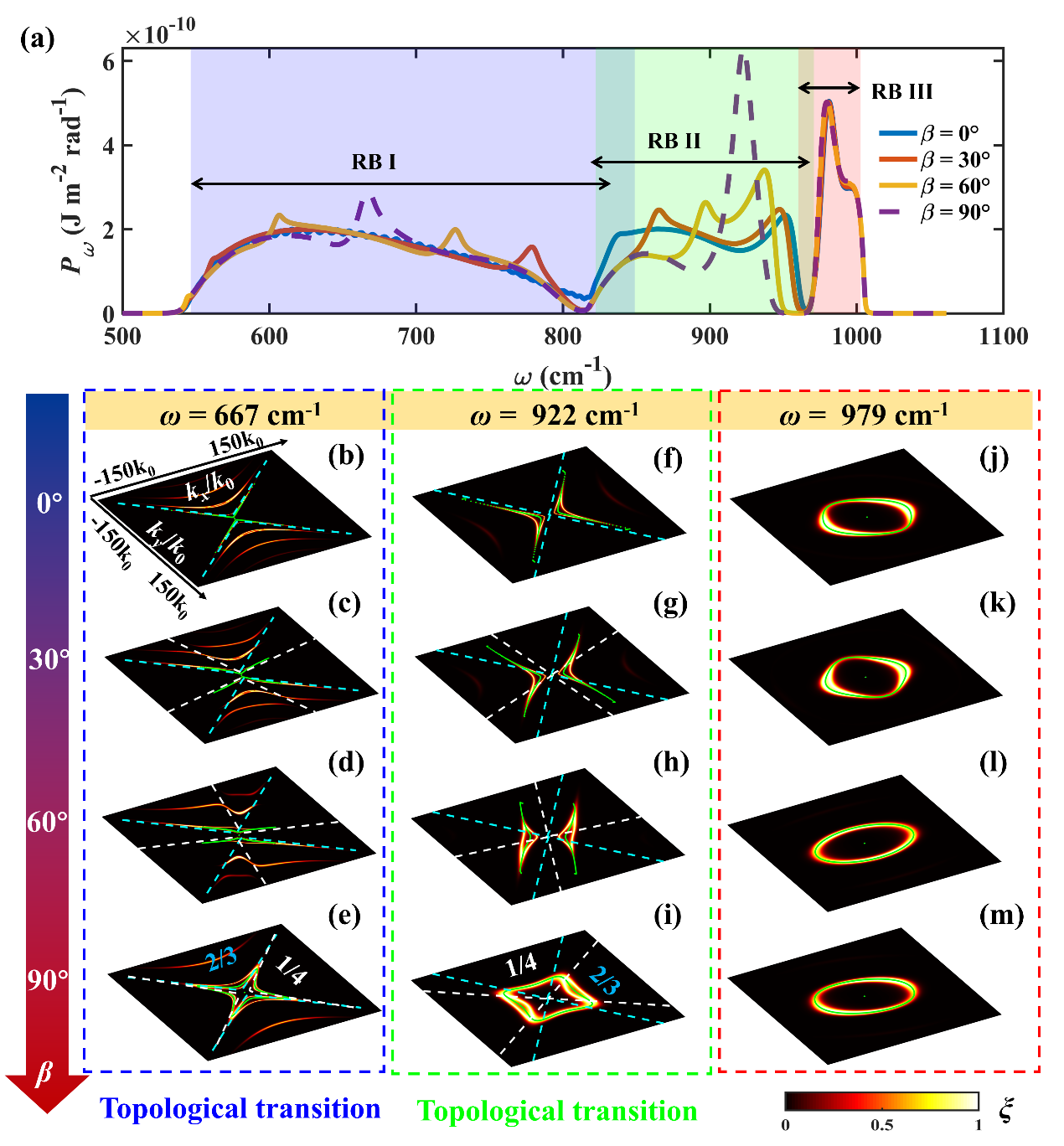


Fig. S6 NFRHT as a function of frequency for twist angles *β* = 0°, *β* = 30°, *β* = 60°, *β* = 90° (blue, red, orange, and violet curves, respectively). The thicknesses *t* of both α-MoO_3_ layers is 50 nm. **(b-m)** Energy transfer coefficient in *k*-space for *β* = 0°, *β* = 30°, *β* = 60°, *β* = 90° (row 1 to 4, respectively) at frequencies *ω* = 667 cm^-1^ **(b-e)**, *ω* = 922 cm^-1^ **(f-i)**, and *ω* = 979 cm^-1^ **(j-m)**. The green lines indicate the IFCs of the bilayer α-MoO_3_. The cyan and white dashed lines indicate the IFCs of the top layer (i.e., without twist) and bottom layer (with twist) of the twisted bilayer α-MoO_3_.

**Section 6. The relative position between the IFC of the bilayer α-MoO_3_ and the bright band of the energy transmission coefficient.**

Figure S7 shows *ξ* in 𝑘-space for different gap distances, along with the IFC of twisted bilayer α-MoO₃ (green lines). At 30 nm and 50 nm, strong coupling between the emitter and reciever leads to symmetric and antisymmetric modes, splitting the fundamental hyperbolic branch into two. The IFC lies between these branches, within the bright band. As the distance increases to 100 nm or 150 nm, the coupling weakens, the bright area in 𝑘-space shrinks, and the mode splitting nearly disappears, with the IFC aligning closer to the bright-band center, indicating weaker coupling and reduced heat-transfer enhancement.


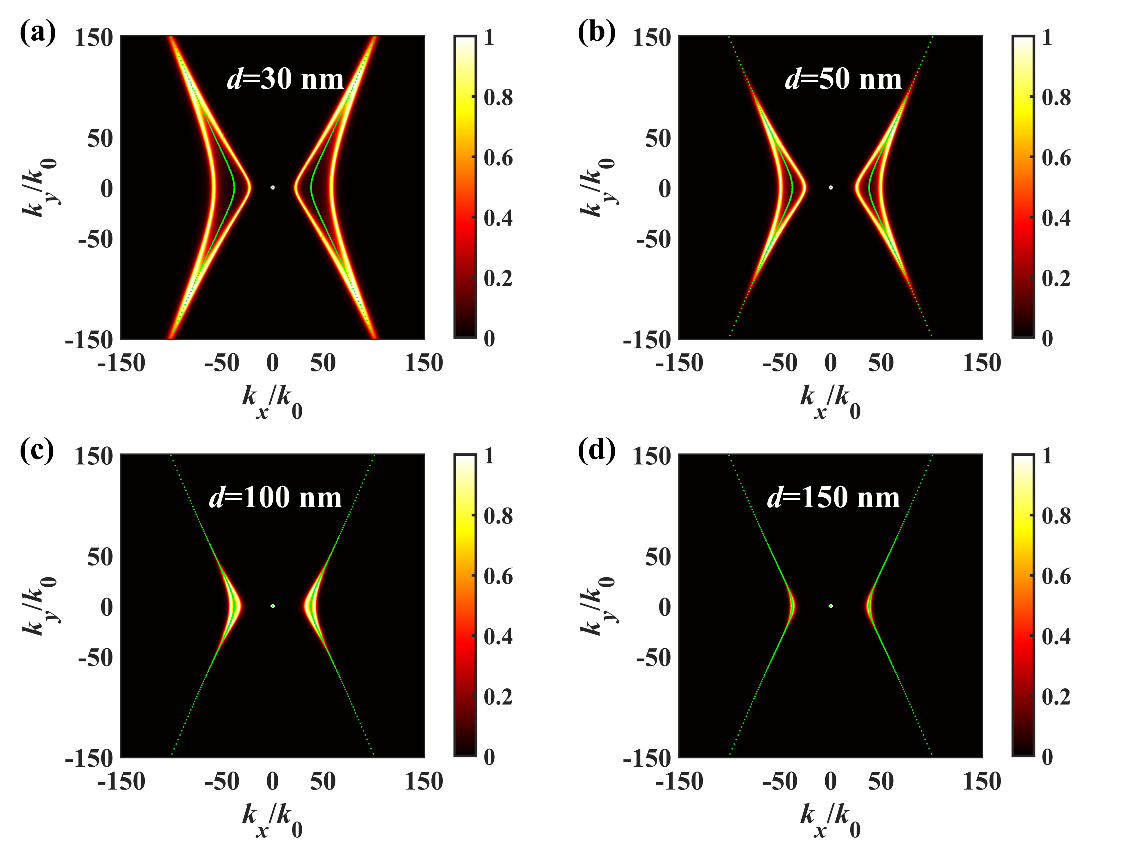


**Fig. S7** Energy transfer coefficient in *k*-space for *d*=30 nm **(a)**, *d*=50 nm **(b)**, *d*=100 nm **(c)**, *d*=150 nm **(d)**. *ω* = 894 cm^-1^, *β* = 0°, *t*=10 nm. The green lines indicate the IFCs of the bilayer α-MoO_3_.
